# Small-Molecule Targeting of VDAC Disrupts Mitochondrial Bioenergetics and Suppresses Melanoma Cell Survival and Migration

**DOI:** 10.3390/cells15121066

**Published:** 2026-06-11

**Authors:** Zhi-Wei Ye, Leilei Zhang, Xuhong Zhang, John Culpepper, Eduardo N. Maldonado, Kenneth D. Tew, Jie Zhang, Danyelle M. Townsend

**Affiliations:** 1 Department of Drug Discovery and Biomedical Sciences, Medical University of South Carolina, 280 Calhoun Street MSC 141, Charleston, SC 29425, USA; tezzh@musc.edu (Z.-W.Y.); maldona@musc.edu (E.N.M.); 2 Department of Pharmacology and Immunology, Medical University of South Carolina, 173 Ashley Avenue BSB 358, Charleston, SC 29425, USA; zhangl@musc.edu (L.Z.); wxzy033@njucm.edu.cn (X.Z.); culpeppj@musc.edu (J.C.); tewk@musc.edu (K.D.T.)

**Keywords:** melanoma, VDAC, mitochondrial bioenergetics, oxidative stress, reactive oxygen species, redox vulnerability, amelanotic melanoma, SC18

## Abstract

**Highlights:**

**What are the main findings?**

- The VDAC-targeting small molecule SC18 disrupts mitochondrial bioenergetics in melanoma cells.
- SC18 induces oxidative stress and impairs melanoma cell survival and migration, with greater sensitivity in low/amelanotic cells.

**What are the implications of the main findings?**

- VDAC-dependent mitochondrial metabolism represents a targetable metabolic vulnerability in melanoma.
- SC18 may serve as a redox-directed metabolic agent with potential for combination therapy.

**Abstract:**

Melanoma is a highly aggressive and metabolically adaptable cancer that often resists conventional therapies. Targeting core bioenergetic pathways may, therefore, represent an effective strategy to improve therapeutic responses, particularly in tumors dependent on mitochondrial function. SC18 is an imidazolidine-2,4-dione compound that binds the NADH-binding pocket of voltage-dependent anion channels (VDACs), inducing mitochondrial dysfunction. VDAC expression is increased in melanoma and strongly associated with advanced disease stage and poor prognosis. In this study, we evaluated the effects of SC18 in melanoma cell lines with distinct pigmentation states, including melanin-rich melanotic human MNT-1 and mouse B16-F1, as well as low/amelanotic human SKMel28 and mouse YUMM cells. VDAC1, VDAC2 and VDAC3 were highly expressed across these melanoma lines, all of which relied on both glycolysis and mitochondrial oxidative phosphorylation for ATP production. SC18 reduced mitochondrial membrane potential and oxygen consumption rates, accompanied by declines in intracellular ATP levels and TCA cycle substrate utilization. SC18 also increased reactive oxygen species, mitochondrial superoxide, and lipid peroxidation, indicating enhanced oxidative stress. These metabolic and redox disturbances were associated with reduced cell viability and significantly impaired migration in multiple melanoma cell lines, supporting a potential anti-metastatic effect. In addition, SC18 showed synergistic cytotoxicity when combined with other chemotherapeutic agents. Overall, SC18 disrupted mitochondrial metabolism, induced oxidative stress, and impaired survival and motility pathways, with more pronounced effects in low/amelanotic than in melanotic melanoma cells. Together, these findings support the further development of SC18 as a mitochondrial metabolic disruptor that targets redox vulnerabilities in melanoma.

## 1. Introduction

Melanoma is the most aggressive form of skin cancer, accounting for over 75% of skin cancer-related deaths despite representing only about 1% of all skin cancer cases [1]. Its incidence continues to rise worldwide, particularly among fair-skinned populations [2]. Although the 5-year survival rate for localized melanoma is as high as 99.6%, it declines sharply to 73.9% in stage III disease and 35.1% in stage IV disease [1], underscoring the major clinical challenge posed by advanced melanoma. Recent advances in immune checkpoint blockade and targeted inhibition of the MAPK pathway, particularly with BRAF and MEK inhibitors, have significantly improved outcomes in melanoma [3,4], compared with the 5-year survival rate of only 15% for stage IV disease in 2015 [1]. However, durable responses remain limited, and approximately 50% of patients eventually fail treatment or experience relapse, especially in metastatic melanoma [3,4]. One factor contributing to this resistance is the marked metabolic plasticity of melanoma cells, which enables them to adapt to diverse microenvironmental and therapeutic stresses by reprogramming energy production and survival pathways [5,6,7,8].

Among the metabolic systems that support melanoma progression, mitochondria play a central role. In addition to generating ATP, mitochondria regulate redox homeostasis, biosynthetic metabolism, and apoptosis, all of which are essential for tumor cell survival and adaptation [9,10,11]. Given the importance of mitochondrial redox balance in these processes, strategies that exploit vulnerabilities in redox pathways may have therapeutic value in melanoma [12,13,14]. Although melanoma has historically been associated with glycolytic metabolism, increasing evidence indicates that many melanoma cells retain substantial mitochondrial respiratory activity and rely on oxidative phosphorylation (OXPHOS) to support proliferation, invasion, metastasis, and therapy resistance [15,16,17,18,19]. Together, these observations suggest that mitochondrial metabolism is not merely a housekeeping process in melanoma, but rather a functionally important and potentially targetable vulnerability.

A key regulator of mitochondrial metabolic exchange is the family of voltage-dependent anion channels (VDACs), which are in the outer mitochondrial membrane. VDACs control the flux of respiratory substrates, adenine nucleotides, and other metabolites between mitochondria and the cytosol, thereby linking mitochondrial bioenergetics with broader cellular metabolism [20,21]. In cancer cells, VDAC conductance can be modulated by endogenous factors such as α/β-tubulin heterodimers and NADH, which may restrict metabolite exchange, suppress mitochondrial metabolism, and help maintain a pro-glycolytic state [22,23,24,25,26]. Of relevance, β-NADH binds to a defined pocket in VDAC1 and sterically reduces pore conductance, resulting in decreased outer membrane permeability to ADP. Because this NADH-binding pocket is conserved across VDAC isoforms, it represents a potentially druggable site for pharmacologic modulation of VDAC gating and mitochondrial metabolite flux [23].

SC18 is a synthetic imidazolidine-2,4-dione derivative designed to occupy the conserved NADH-binding pocket on the inner wall of VDAC isoforms [23]. Prior work has shown that SC18 modulates NADH-dependent channel regulation, decreases mitochondrial membrane potential, and induces cell death in hepatocarcinoma cells [23,24]. These findings raise the possibility that SC18 may also disrupt mitochondrial metabolism in melanoma, a tumor type characterized by pronounced metabolic heterogeneity and redox adaptability [14,15,16,17,18,19]. Pigmentation status is closely associated with distinct metabolic and redox states in melanoma; reduced pigmentation has been linked to elevated oxidative stress, diminished antioxidant buffering, impaired ATP production, and suppression of glycolysis, oxidative phosphorylation, and pentose phosphate pathway flux [12,14,27,28]. Accordingly, melanotic and amelanotic melanoma cells may differ in their reliance on mitochondrial function and in their susceptibility to VDAC-directed metabolic stress.

In the present study, we investigated the effects of SC18 in melanotic melanoma cell lines (human MNT-1 and mouse B16) and amelanotic melanoma cell lines (human SKMel28 and mouse YUMM). We sought to determine whether targeting VDAC with SC18 alters mitochondrial bioenergetics and redox homeostasis in melanoma cells and whether responses differ across pigmentation-associated metabolic phenotypes. By addressing these questions, this study aims to evaluate VDAC-dependent mitochondrial metabolism as a potential therapeutic vulnerability in melanoma.

## 2. Materials and Methods

### 2.1. Cell Lines and Reagents (Table A1)

B16-F1, YUMM, MNT-1, and SKMel28 melanoma cell lines were used in this study. B16-F1, YUMM, and MNT-1 cells were obtained from ATCC (Manassas, VA, USA). SKMel28 cells were a kind gift from Prof. Philip H. Howe (Medical University of South Carolina).

B16-F1 cells were maintained in Dulbecco’s modified Eagle’s medium (DMEM; Corning, Manassas, VA, USA) supplemented with 10% fetal bovine serum (FBS; Atlanta Biologicals, Flowery Branch, GA, USA) and 1× penicillin–streptomycin (Corning). YUMM cells were cultured in DMEM/F-12 50/50 (Corning, Manassas, VA, USA) supplemented with 10% FBS (Atlanta Biologicals), 1× MEM nonessential amino acids (Corning), and 1× penicillin–streptomycin (Corning). MNT-1 melanoma cells were maintained in DMEM (Corning) supplemented with 20% FBS, 10% AIM-V medium (Gibco, Grand Island, NY, USA), 1× MEM non-essential amino acids (Corning), and 1× penicillin–streptomycin. SKMel28 cells were cultured in RPMI 1640 medium (Corning) supplemented with 10% FBS and 1× penicillin–streptomycin. All cells were maintained at 37 °C in a humidified atmosphere with 5% CO_2_.

### 2.2. Method Details

#### 2.2.1. VDAC Expression and Survival Analysis Using GEPIA2

mRNA expression and survival analyses for VDAC1, VDAC2, and VDAC3 were generated from the GEPIA2 web server (http://gepia2.cancer-pku.cn). Skin cutaneous melanoma (SKCM) tumor (n = 461) and normal tissue (n = 558) datasets were queried to compare expression in tumors versus normal tissues and across clinical stages (Stage Plot module). Overall survival was evaluated using the Survival Analysis module, with patients stratified into high- and low-expression groups based on median VDAC expression. Expression and survival plots were generated and exported directly from GEPIA2.

#### 2.2.2. VDAC Expression in Melanoma Cells by Western Blot Analysis

Total soluble protein from melanoma cells was quantified using a BCA protein assay (Thermo Fisher, Waltham, MA, USA), and equal amounts of protein were separated by SDS-PAGE (Bio-Rad, Hercules, CA, USA) and transferred onto low-fluorescence PVDF membranes (Sigma, Burlington, MA, USA) using a Trans-Blot Turbo Transfer System (Bio-Rad). Membranes were incubated overnight at 4 °C with primary antibodies listed in the Key Resources Table. Immunoblots were then incubated with IRDye-conjugated secondary antibodies, imaged using a two-channel (700/800 nm) Odyssey CLx near-IR fluorescence imaging system (LI-COR, Lincoln, NE, USA), and quantified with Image Studio v4.0 software (LI-COR).

### 2.3. Cellular Melanin Determination

Melanin-containing pellets collected from melanoma cell lysates were washed with PBS and solubilized by boiling in 1 M NaOH containing 10% DMSO for 15 min. The absorbance of the dissolved melanin solution was measured at 470 nm. Melanin concentrations were calculated using a standard curve generated with synthetic melanin (MP Biomedicals, Solon, OH, USA) over a range of 7.81–500 µg/mL using a CLARIOstar microplate reader (BMG LABTECH, Cary, NC, USA).

### 2.4. Cytotoxicity Assay

Melanoma cells were seeded in 96-well plates and treated with the indicated concentrations of SC18 for 240 h in 0.1% FBS medium. Cell viability was assessed by MTT assay. For combination treatments with anticancer drugs, cells were seeded in 96-well plates and treated for 48 h with the indicated concentrations of doxorubicin (Sigma), PABA/NO (provided by Dr. Larry Keefer, NCI at Frederick), paclitaxel (Selleck Chemicals, Houston, TX, USA), ME344 (MEI Pharma, San Diego, CA, USA), or vinblastine (Sigma) in the presence or absence of SC18. MTT reagent (Sigma; 0.5 mg/mL) was then added and incubated for 4 h, followed by the addition of solubilization solution (10% SDS in 0.1 M HCl). Absorbance was measured at 550 and 690 nm using a CLARIOstar microplate reader.

### 2.5. Detection of Total Reactive Oxygen Species (ROS), Mitochondrial ROS, Lipid ROS, and Mitochondrial Membrane Potential

Total ROS, mitochondrial ROS, lipid ROS, and mitochondrial membrane potential were assessed using flow cytometry. Cells were incubated with 1 μM 2′,7′-dichlorodihydrofluorescein diacetate (H_2_DCFDA, Thermo Fisher) for total ROS, 1 μM MitoSOX (Thermo Fisher) for mitochondrial ROS, 500 nM BODIPY 665 (Thermo Fisher) for lipid ROS, and 200 nM Tetramethylrhodamine (TMRM, Sigma) for mitochondrial membrane potential for 30 min, followed by two washes with PBS. Fluorescent signals were acquired on a Beckman CytoFLEX S flow cytometer and analyzed using CytExpert v2.1 software (Beckman Coulter, Indianapolis, IN, USA) in the Analytical Redox Core.

### 2.6. Mitochondrial Function Using Phenotype MicroArray MitoPlate S-1

Mitochondrial substrate utilization was profiled using MitoPlate S-1 plates (Biolog, Hayward, CA, USA), which contain tricarboxylic acid (TCA) cycle intermediates, amino acids, and ketone bodies spotted in triplicate. Cytosolic substrates (D-glucose, glycogen, D-glucose 1-phosphate, D-glucose 6-phosphate, 6-phospho-D-gluconate, and lactate) on the MitoPlate S-1 served as negative controls. Pyruvate and selected mitochondrial substrates (L-leucine, γ-aminobutyric acid, α-ketoisocaproic acid, acetylcarnitine, octanoylcarnitine, and palmitoylcarnitine) were also tested in the presence of “sparker” malate (100 μM).

Substrates were dissolved by incubating the plates with 30 μL of Assay Mix (2× BMAS, 6× Redox Dye MC, and saponin at 30 μg/mL for cell permeabilization) with or without SC 18 in a 5% CO_2_ incubator at 37 °C for 1 h. Melanoma cells (40,000 per well) were then added in 30 μL to each well. Plates were immediately transferred to the reader, and kinetic changes in dye reduction were recorded in real time as described above. For each substrate, signals from the three technical replicates were averaged. Metabolic activity was quantified by calculating the area under the curve (AUC) for total substrate utilization and the initial rate to assess the early velocity of substrate metabolism.

### 2.7. Seahorse XF ATP Rate Assay

An Agilent Seahorse XF Pro Analyzer (Agilent Technologies, Santa Clara, CA, USA) was used to assess mitochondrial bioenergetics and ATP production in melanoma cells under control conditions and after 24 h of SC18 treatment. Cells were seeded directly into Agilent Seahorse XF Pro M cell culture microplates and allowed to adhere for 24 h, after which the growth medium was replaced with Seahorse XF assay medium lacking bicarbonate and supplemented according to the manufacturer’s instructions. A real-time ATP rate assay was performed on identically treated plates using the same analyzer and assay medium, but without FCCP. OCR and extracellular acidification rate (ECAR) were recorded at baseline and after sequential addition of oligomycin A followed by rotenone/antimycin A, and mitochondrial versus glycolytic ATP production rates were calculated using the Agilent ATP Rate Assay protocol. Each measurement cycle consisted of a 2 min mixing period followed by a 3 min measurement period. Three baseline cycles were recorded prior to the first injection, followed by three cycles after each subsequent injection. Each condition was tested in 12 replicate wells in three independent experiments.

### 2.8. Annexin V/PI Apoptosis Assay

Apoptosis was assessed using Annexin V and propidium iodide (PI) staining (San Diego, CA, USA) followed by flow cytometry. Briefly, melanoma cells were seeded in culture plates and treated with vehicle control or 25 µM SC18 for 24 h. After treatment, both floating and adherent cells were collected to avoid loss of apoptotic or dead cell populations. Adherent cells were gently detached, combined with the corresponding floating cells, washed with cold PBS, and resuspended in Annexin V binding buffer. Cells were then stained with Annexin V and PI according to the manufacturer’s instructions and incubated for 15 min at room temperature in the dark. Samples were analyzed by flow cytometry using a CytoFLEX flow cytometer. Unstained cells and single-stained controls were used to set compensation and gates. Cell populations were classified as viable cells (Annexin V^−^/PI^−^), early apoptotic cells (Annexin V^+^/PI^−^), late apoptotic/dead cells (Annexin V^+^/PI^+^), and necrotic/dead cells (Annexin V^−^/PI^+^). Quantification of apoptosis was calculated as the percentage of total Annexin V-positive cells, defined as the sum of Annexin V^+^/PI^−^ and Annexin V^+^/PI^+^ populations. Data were obtained from three independent experiments.

### 2.9. Caspase-3 Activity Assay

Caspase-3 activity was assessed using the EnzChek Caspase-3 Assay Kit #2 (Molecular Probes/Invitrogen, Eugene, OR, USA). Briefly, melanoma cells were lysed following treatment, and equal amounts of lysate protein were incubated in a caspase reaction buffer containing 10 mM PIPES, pH 7.4, 2 mM EDTA, 0.1% CHAPS, 5 mM DTT, and 25 μM Z-DEVD-R110. Cleavage of Z-DEVD-R110 releases fluorescent rhodamine 110 (R110), which was monitored using a CLARIOstar microplate reader at excitation/emission wavelengths of 496/520 nm. R110 standards were prepared in caspase reaction buffer using serial 2-fold dilutions from 1 μM to 1 nM. Caspase-3 activity was normalized to lysate protein content.

### 2.10. Wound Healing Assay

Cell migration was assessed using a scratch (wound healing) assay. Melanoma cells were seeded into 6-well plates and grown to 90–100% confluence as described previously [13]. A uniform linear scratch was created in the cell monolayer using a sterile yellow pipette tip, and detached cells were removed by gently washing twice with PBS. Cells were then incubated in complete medium containing 25 μM SC18 (or vehicle control). Phase-contrast images of the wound area were acquired immediately after scratching (0 h) and after 24 h using an EVOS M7000 Imaging System, with three fields imaged per well. Wound closure was evaluated by comparing the remaining cell-free area at 24 h relative to 0 h.

### 2.11. Quantification and Statistical Analysis

Statistical analyses were performed with GraphPad Prism v8 (GraphPad, La Jolla, CA, USA) or Student’s two-tailed *t* tests. *, **, and *** indicate *p* < 0.05, *p* < 0.005, and *p* < 0.0001, respectively. Data were expressed as mean ± SD from three independent experiments.

## 3. Results

### 3.1. VDAC Expression Is Elevated in Skin Cutaneous Melanoma and Is Associated with Stage and Patient Outcome

**Figure 1:**
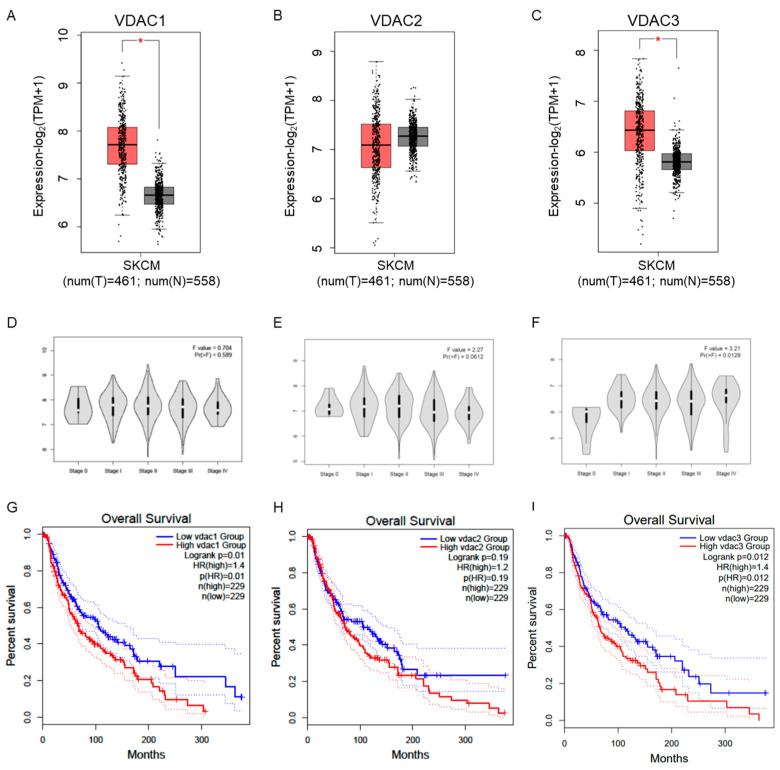
VDAC isoform expression is elevated in melanoma and is associated with poor patient survival. mRNA expression and survival analyses of VDAC1, VDAC2, and VDAC3 were performed using the GEPIA2 web server. The TCGA/GTEx SKCM datasets (tumor, n = 461; normal, n = 558) were used to compare tumor and normal expression levels (Box Plot) and to assess expression across clinical stages (Stage Plot). Survival analyses were conducted using the Survival Analysis module, with patients stratified into high- and low-expression groups based on the median expression level. (**A**–**C**) Tumor versus normal expression of VDAC1–3. Expression levels are presented as log2 (TPM + 1), where TPM indicates transcripts per million. The |Log2FC| cutoff was set at 0.585, corresponding to a fold change > 1.5, and the *p*-value cutoff was set at 0.001 (*, *p* < 0.001). (**D**–**F**) Stage-dependent expression for VDAC1-3 with ANOVA *p* values shown. (**G**–**I**) Kaplan–Meier survival plots (overall survival for VDAC1-3) with log-rank *p* values and hazard ratios (HR) reported. Plots were exported directly from GEPIA2.

Using the GEPIA2 platform, we queried the TCGA/GTEx SKCM datasets to examine mRNA expression profiles of VDAC1, VDAC2, and VDAC3 in tumor versus normal tissues and across clinical stages. Compared with normal skin, VDAC1 and VDAC3 were significantly upregulated in SKCM tumors (*p* < 0.001), whereas VDAC2 showed no significant difference (Figure 1A–C). Stage-wise analysis suggested a progression-related pattern. VDAC3 expression increased significantly across stages (ANOVA, *p* = 0.0129), whereas VDAC2 showed a decreasing trend (ANOVA, *p* = 0.0612), and VDAC1 did not vary significantly by stage (ANOVA, *p* = 0.589) (Figure 1D–F).

We next evaluated prognostic relevance by stratifying patients into high- and low-expression groups based on the median expression level. High VDAC1 expression was associated with worse overall survival (log-rank *p* = 0.01; HR (high) = 1.4). VDAC2 expression was not significantly associated with overall survival (log-rank *p* = 0.19; HR (high) = 1.2). High VDAC3 expression correlated with poorer overall survival (log-rank *p* = 0.012; HR (high) = 1.4) (Figure 1G–I). Collectively, these analyses indicated that VDAC1 and VDAC3 are most consistently associated with tumor enrichment and adverse clinical outcomes in SKCM, suggesting that VDAC-associated pathways may contribute to melanoma progression.

### 3.2. Melanoma Models Show Robust but Isoform-Specific VDAC Expression

**Figure 2:**
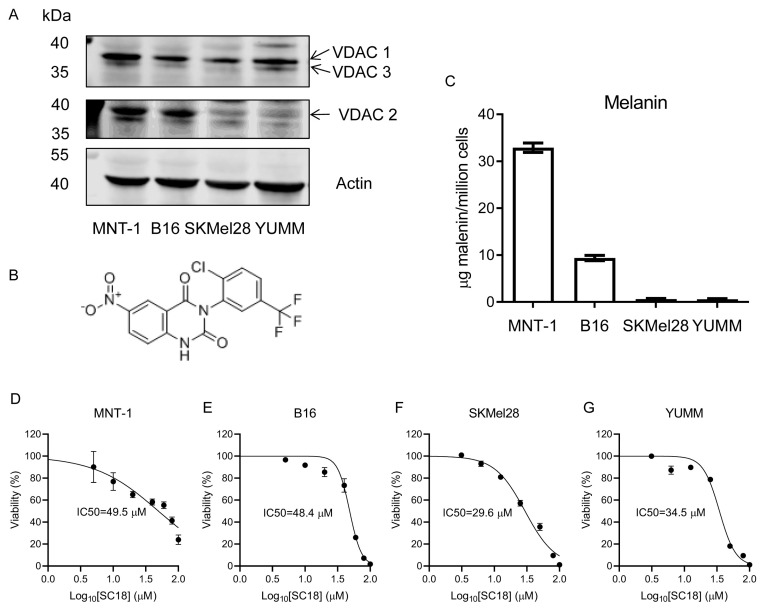
Melanoma cells exhibit high VDAC expression; SC18 treatment reduces melanoma cell viability. (**A**) Protein levels of VDAC1, VDAC2, and VDAC3 were assessed by immunoblotting in MNT-1, B16, SKMel28, and YUMM cells; β-actin served as a loading control. (**B**) Chemical structure of SC18 (1-(4-chloro-2-fluoroethynylphenyl)-3-(2-nitrophenyl) imidazolidine-2,4-dione dihydrofluoride), a VDAC inhibitor that targets the NADH-binding pocket of VDAC. (**C**) Melanin content of melanoma cells. (**D**–**G**) Cytotoxic effects of SC18 on MNT-1, B16, SKMel28, and YUMM cells treated with increasing concentrations of SC18 for 240 h. Cell viability was measured by MTT assay, and absorbance at 570 nm was normalized to vehicle-treated controls. Values are relative to vehicle controls and represent mean ± S.D. of three independent experiments.

To establish clinically relevant melanoma models for interrogating VDAC biology and pharmacologic targeting, we profiled baseline VDAC isoform expression in two human melanoma cell lines (MNT-1 and SKMel28) and two murine melanoma cell lines (B16 and YUMM). Immunoblotting detected VDAC1, VDAC2, and VDAC3 in all four models (Figure 2A), confirming that the VDAC axis is broadly present across both species. Notably, while VDAC1 and VDAC3 levels were generally comparable across cell lines, VDAC2 exhibited pronounced cell line-dependent variation, with higher VDAC2 abundance in melanotic MNT-1 and B16 relative to low/amelanotic SKMel28 and YUMM. Consistent with their pigmentation status, melanin content was highest in MNT-1 cells, intermediate in B16 cells, and nearly undetectable in SKMel28 and YUMM cells (Figure 2C). Together, these data indicate that melanoma models differ in their baseline expression of specific VDAC isoforms, with VDAC2 showing an apparent association with melanin-producing melanoma phenotypes.

We next tested whether SC18 (Figure 2B) reduced cell survival. Cells were treated with increasing concentrations of SC18 for 240 h, and viability was quantified by MTT assay (Figure 2D–G). Across all four melanoma models, SC18 reduced cell viability in a concentration-dependent manner, with IC_50_ values ranging from approximately 29 to 50 μM. Low-pigment/amelanotic melanoma cells (SKMel28, IC_50_ = 29.6 μM; YUMM, IC_50_ = 34.5 μM) were more sensitive than melanotic lines (MNT-1, IC_50_ = 49.5 μM; B16, IC_50_ = 48.4 μM) under the same dosing regimen, suggesting that pigmentation status may influence susceptibility to VDAC-targeted stress (Figure 2).

### 3.3. SC18 Suppresses Mitochondrial ATP Production and Triggers Compensatory Glycolysis, Causing Net ATP Depletion

**Figure 3:**
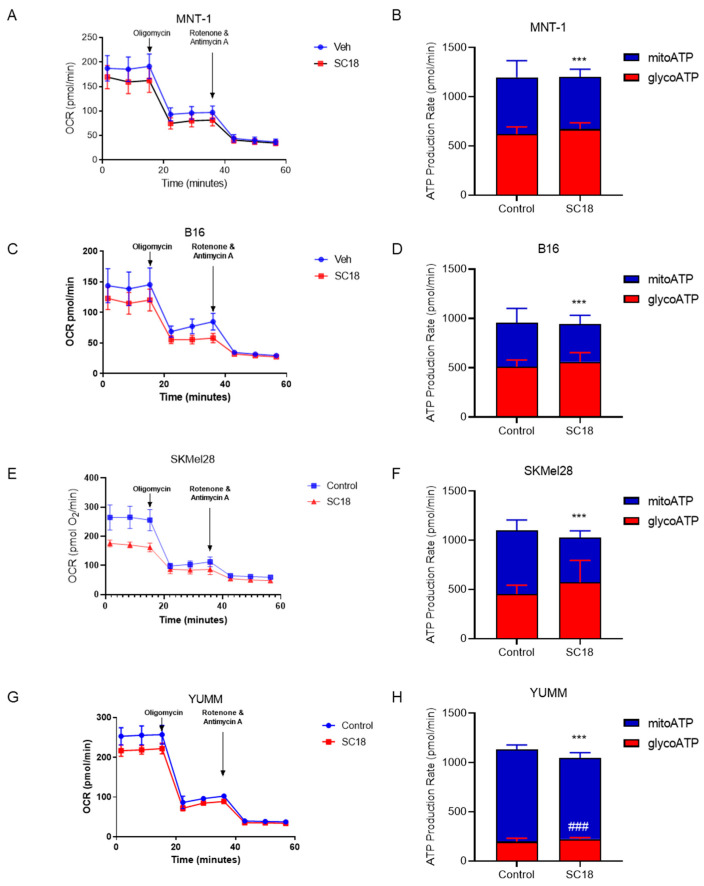
Melanoma cells rely on mitochondrial oxidative phosphorylation (OXPHOS), and SC18 suppresses OXPHOS while promoting compensatory glycolysis. SC18 reduced mitochondrial-derived ATP and increased glycolytic ATP as a compensatory response, resulting in overall ATP depletion, particularly notable in amelanotic cells. ATP production from glycolysis and OXPHOS was simultaneously quantified using the Seahorse XF Real-Time ATP Rate Assay with sequential injection of oligomycin and rotenone/antimycin A in (**A**,**B**) MNT-1, (**C**,**D**) B16, (**E**,**F**) SKMel28, and (**G**,**H**) YUMM cells. Data are represented as mean ± S.D. of three independent experiments. ***, *p* < 0.001 vs. vehicle control in mitoATP; ^###^, *p* < 0.001 vs. vehicle control in glycoATP by Student’s *t* test.

To define baseline bioenergetic states and determine how SC18 perturbed ATP production, we quantified mitochondrial (OXPHOS-derived) ATP and glycolytic ATP using the Seahorse XF Real-Time ATP Rate Assay in human MNT-1 and SKMel28 cells and murine B16 and YUMM cells. In vehicle controls, all four cell lines generated ATP from both pathways (Figure 3). However, the relative contribution of mitochondrial ATP was higher in low/amelanotic cells (SKMel28 and especially YUMM) (Figure 3E–H) compared with melanotic MNT-1 and B16 (Figure 3A–D), indicating a more OXPHOS-weighted baseline phenotype in these cells.

Upon SC18 treatment, mitochondrial ATP production decreased across all lines, consistent with suppression of OXPHOS. In parallel, glycolytic ATP increased, indicating a compensatory shift toward glycolysis (Figure 3). Despite this, total ATP output (mitochondrial + glycolytic ATP) declined, showing that glycolytic up-regulation was insufficient to offset the loss of mitochondrial ATP. This net ATP depletion was most pronounced in the low/amelanotic models, with YUMM showing the strongest overall reduction (Figure 3E–H), consistent with heightened energetic vulnerability to SC18-mediated OXPHOS inhibition.

### 3.4. MitoPlate S-1 Profiling in Permeabilized Melanoma Cells Reveals Pigmentation-Associated Substrate Oxidation and Broader SC18 Sensitivity in Amelanotic Models

To interrogate mitochondrial function and substrate utilization, we employed the Biolog (Odin) MitoPlate S-1 assay, a cell-based phenotyping platform that uses 96-well plates pre-loaded with a diverse panel of mitochondrial substrates (and, in related formats, inhibitors). Cells of interest are added to the wells together with a proprietary redox dye, and substrate-driven mitochondrial metabolism generates energy-rich NADH that reduces the dye, producing a colorimetric signal quantified by the Odin™ reader. In our experiments, cells were permeabilized to minimize plasma-membrane transport limitations and enable more direct assessment of mitochondrial substrate oxidation.

**Figure 4:**
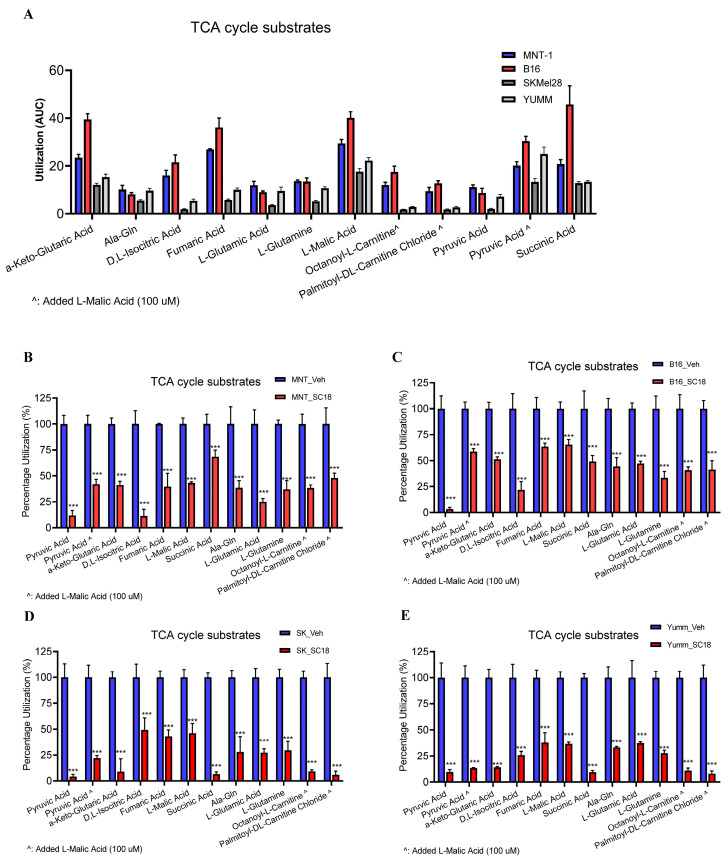
Metabolic reprogramming induced by SC18 in YUMM cells. SC18 reduces the utilization of TCA cycle substrates in YUMM melanoma cells. Substrate oxidation profiles in YUMM cells were assessed using the Odin Mito S1 plate. Mitochondrial substrate utilization was assessed using Biolog MitoPlates™ (MitoPlate S-1), a cell-based mitochondrial phenotyping assay performed in 96-well plates pre-loaded with a diverse panel of mitochondrial substrates. (**A**) Substrates prioritized from the screen based on robust utilization signals across the panel; data are expressed as AUC. (**B**–**E**) Substrate utilization profiles in (**B**) MNT-1, (**C**) B16, (**D**) SKMel28, and (**E**) YUMM, highlighting SC18-sensitive substrates spanning pyruvate-supported oxidation (pyruvate ± L-malic acid, 100 μM), TCA anaplerotic substrates (α-ketoglutarate, D,L-isocitrate, fumarate, L-malic acid, succinate), glutamine/glutamate and Ala–Gln, and carnitine-linked fatty-acid substrates (octanoyl-L-carnitine and palmitoyl-DL-carnitine; with L-malic acid supplementation as indicated). Data are presented as mean ± S.D. from three independent experiments and expressed as percentage of vehicle for each cell line. ***, *p* < 0.001 vs. vehicle control by Student’s *t* test.

For all melanoma cell lines, we first identified a set of substrates with robust utilization signals, including pyruvate (±L-malic acid, 100 μM), TCA/anaplerotic substrates (α-ketoglutarate, D,L-isocitrate, fumarate, L-malic acid, succinate), glutamine/glutamate and Ala-Gln, and carnitine-linked fatty-acid substrates (octanoyl-L-carnitine and palmitoyl-DL-carnitine; with L-malic acid supplementation as indicated). Baseline profiling showed that melanotic cells exhibited higher overall dye-reduction responses for these substrates than the amelanotic models, consistent with greater substrate-supported mitochondrial oxidative capacity in pigmented melanoma cells (Figure 4A).

We then assessed the impact of VDAC inhibition (25 μM SC18 for 24 h) prior to MitoPlate analysis. SC18 caused broad suppression of substrate-supported mitochondrial activity across multiple pathways, including pyruvate-supported oxidation (pyruvate ± malate), TCA cycle–linked oxidation (α-ketoglutarate, isocitrate, fumarate, malate, succinate), glutamine/glutamate/Ala–Gln–supported inputs, and carnitine-linked fatty-acid substrates (octanoyl- and palmitoyl-carnitine). Of note, inhibition was more pronounced in amelanotic cells, with YUMM showing the most global reduction across the profiled substrates, whereas melanotic MNT-1 and B16 retained comparatively higher residual substrate utilization after SC18 (Figure 4B–D). Together, these findings indicate that SC18 broadly constrains mitochondrial substrate oxidation, with enhanced metabolic vulnerability in amelanotic cells.

### 3.5. SC18 Depolarizes Mitochondria and Enhances Mitochondrial Superoxide, with Stronger MitoSOX Induction in Amelanotic Melanoma Cells

**Figure 5:**
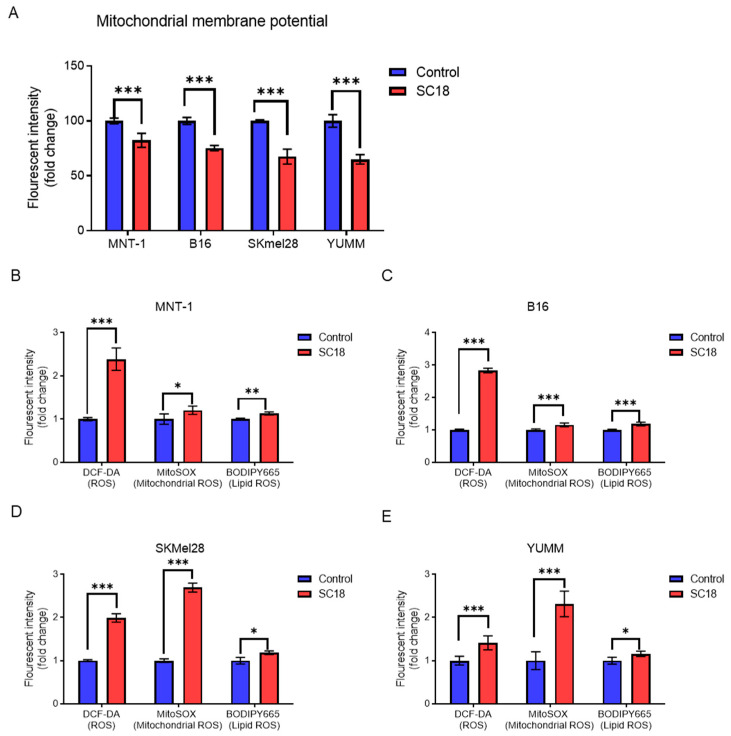
SC18 disrupts mitochondrial membrane potential and induces oxidative stress in melanoma cells. (**A**) Mitochondrial membrane potential (Δψm) was measured in MNT-1, B16, SKMel28, and YUMM cells using TMRM staining followed by flow cytometry after 24 h of SC18 treatment. (**B**–**E**) Oxidative stress markers were measured in MNT-1 (**B**), B16 (**C**), SKMel28 (**D**), and YUMM (**E**) cells following SC18 treatment. Intracellular ROS was assessed using DCF-DA staining, mitochondrial superoxide was measured using MitoSOX staining, and lipid peroxidation was determined using BODIPY 665 staining, followed by flow cytometric analysis. Values are expressed relative to vehicle controls and represent the mean ± S.D. of three independent experiments. *, *p* < 0.05; **, *p* < 0.01; ***, *p* < 0.001 versus vehicle control by Student’s *t* test.

To assess whether SC18 perturbs mitochondrial integrity and redox homeostasis, we quantified mitochondrial membrane potential (Δψm) and multiple oxidative stress markers by flow cytometry after 24 h of treatment in MNT-1, B16, SKMel28, and YUMM cells (Figure 5). Mitochondrial membrane potential was measured using TMRM staining, a potentiometric dye commonly used to monitor Δψm. Across all four melanoma cell lines, SC18 significantly reduced TMRM fluorescence, indicating loss of Δψm and mitochondrial depolarization (Figure 5A).

We next evaluated whether SC18-induced mitochondrial depolarization was accompanied by increased oxidative stress. SC18 increased intracellular ROS, as measured by DCF-DA, and lipid peroxidation, as measured by BODIPY 665, with responses that were generally comparable among the four melanoma cell lines (Figure 5B–E). In contrast, mitochondrial superoxide accumulation, measured by MitoSOX, showed a pigmentation-associated pattern. The low/amelanotic SKMel28 and YUMM cells exhibited a more pronounced increase in MitoSOX fluorescence following SC18 treatment compared with the melanotic MNT-1 and B16 cells. Together, these data demonstrate that SC18 compromises mitochondrial membrane potential and promotes oxidative stress in melanoma cells. Notably, SC18-induced mitochondrial superoxide accumulation was greater in low/amelanotic melanoma cells, suggesting that pigmentation status may influence the redox response to mitochondrial disruption by SC18.

### 3.6. SC18 Induces Apoptosis in Melanoma Cells

**Figure 6:**
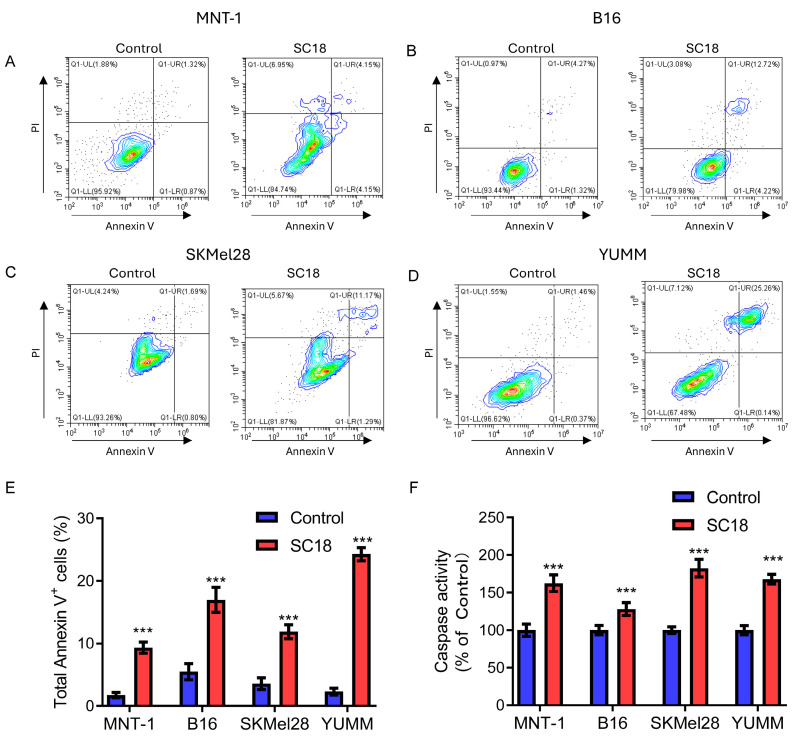
SC18 induces apoptosis in melanoma cells. Representative Annexin V/PI flow cytometry plots of (**A**) MNT-1, (**B**) B16, (**C**) SKMel28, and (**D**) YUMM cells treated with vehicle control or SC18. Cell populations were classified as viable cells (Annexin V^−^/PI^−^), early apoptotic cells (Annexin V^+^/PI^−^), late apoptotic/dead cells (Annexin V^+^/PI^+^), and necrotic/dead cells (Annexin V^−^/PI^+^). (**E**) Quantification of total Annexin V-positive cells, calculated as the sum of Annexin V^+^/PI^−^ and Annexin V^+^/PI^+^ populations, from three independent experiments. (**F**) Caspase-3 activity was measured in all four melanoma cell lines using the Z-DEVD-R110 fluorogenic substrate assay. Data are presented as mean ± S.D. *** *p* < 0.001 versus vehicle control.

Given that SC18 disrupted mitochondrial function and altered mitochondrial membrane potential, we next examined whether SC18 treatment promoted apoptotic cell death in melanoma cells. Apoptosis was assessed by Annexin V/PI staining followed by flow cytometry in MNT-1, B16, SKMel28, and YUMM cells treated with vehicle control or SC18. Representative flow cytometry plots showed an increase in Annexin V-positive cell populations following SC18 treatment across all four melanoma cell lines (Figure 6A–D). Quantitative analysis from three independent experiments demonstrated that SC18 significantly increased the percentage of total Annexin V-positive cells, calculated as the sum of early apoptotic cells (Annexin V^+^/PI^−^) and late apoptotic/dead cells (Annexin V^+^/PI^+^), compared with vehicle-treated controls (Figure 6E). Consistent with the differential sensitivity observed in previous functional assays, the increase in Annexin V-positive cells was more pronounced in the low/amelanotic SKMel28 and YUMM cells than in the melanotic MNT-1 and B16 cells.

To further validate apoptosis induction, caspase-3 activity was measured in all four melanoma cell lines using a fluorogenic Z-DEVD-R110 substrate assay. Consistent with the Annexin V/PI flow cytometry results, SC18 treatment increased caspase-3 activity across all four cell lines, with a stronger response observed in SKMel28 and YUMM cells compared with MNT-1 and B16 cells (Figure 6F). Together, these findings indicate that SC18 promotes apoptotic cell death in melanoma cells and that low/amelanotic melanoma cells exhibit greater apoptotic sensitivity to SC18 treatment.

### 3.7. SC18 Suppresses Melanoma Cell Migration

**Figure 7:**
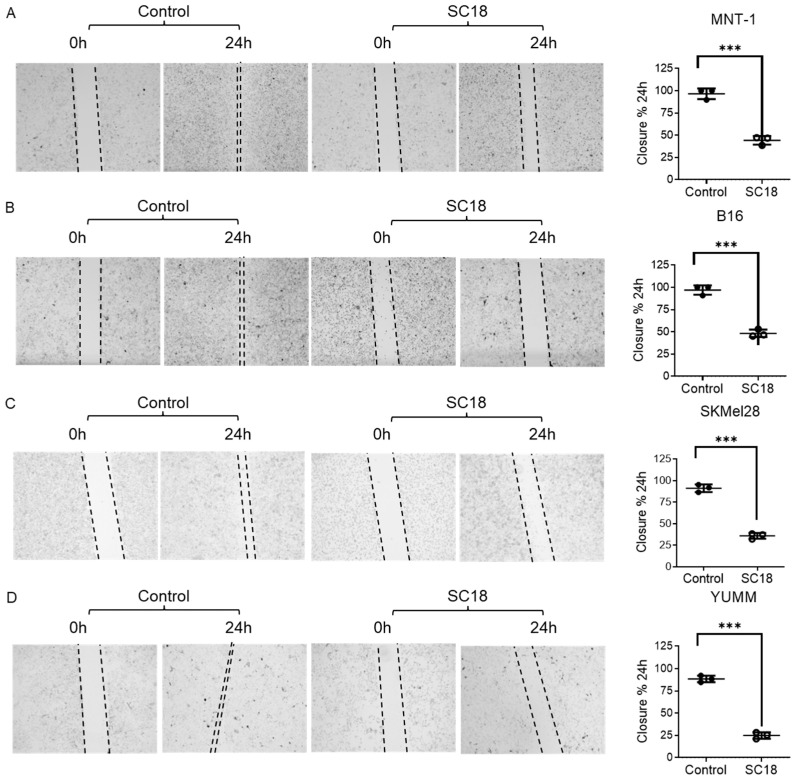
SC18 suppresses melanoma cell migration in a wound healing assay. Representative phase-contrast images of scratch wounds at 0 and 24 h in (**A**) MNT-1, (**B**) B16, (**C**) SKMel28, and (**D**) YUMM cells treated with vehicle or 50 μM SC18. Wound closure was quantified using ImageJ software (Version 1.54p) based on wound area and expressed as percentage closure relative to the 0-h time point. Values are relative to vehicle controls and represent the mean ± S.D. of three independent experiments. ***, *p* < 0.001 vs. vehicle control by Student’s *t* test.

To assess the impact of SC18 on melanoma cell motility, we performed wound-healing assays in each of the cell lines, monitoring wound closure over 24 h. In vehicle-treated controls, cells migrated progressively into the scratched area, resulting in steady gap narrowing over time. In contrast, SC18-treated cells had a clear delay in gap closure in all cell lines, with visibly larger residual wound areas remaining at 24 h, indicating impaired migratory capacities (Figure 7).

Quantification of percentage wound closure confirmed significant reductions in motility following SC18 treatment in each cell line and revealed consistent pigmentation-associated differences in sensitivity. Specifically, amelanotic cells were more responsive to SC18-mediated migration inhibition, as SKMel28 and YUMM (Figure 6C,D) showed a greater reduction in wound closure than the melanotic lines MNT-1 and B16 (Figure 6A,B). Together, these data demonstrate that SC18 suppresses melanoma cell migration in a 2D wound-healing setting, with enhanced sensitivity in amelanotic melanoma cells, most notably YUMM, suggesting that pigmentation status may influence response to SC18-associated stress.

### 3.8. SC18 Enhances the Cytotoxic Efficacy of Multiple Anti-Tumor Agents in Melanoma Cells

**Figure 8:**
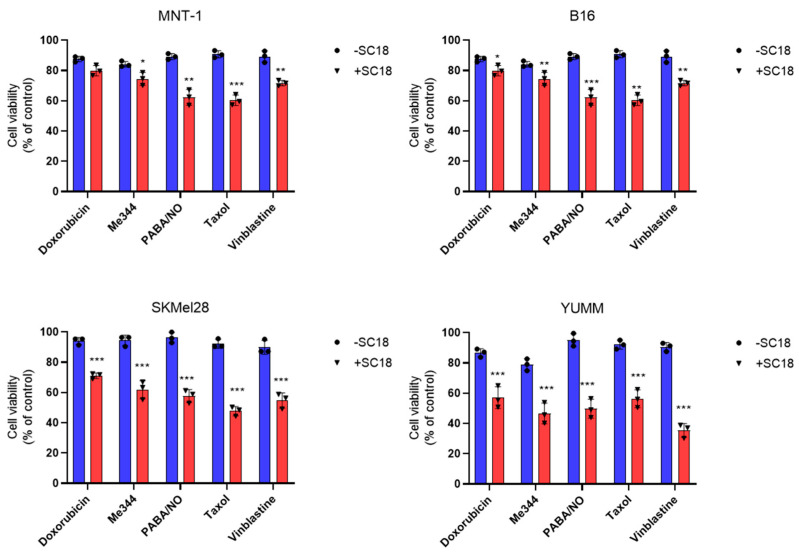
SC18 enhances the cytotoxic efficacy of multiple anti-tumor agents in melanoma cells. Human melanotic MNT-1 and murine melanotic B16 cells, as well as human amelanotic SKMel28 and murine amelanotic YUMM cells, were treated with doxorubicin (2 μM), Me344 (10 μM), PABA/NO (10 μM), paclitaxel (Taxol, 1 μM), or vinblastine (2 μM) in the absence (-SC18) or presence (+SC18) of SC18 (5 μM). Cell viability was assessed by MTT assay, and absorbance at 570 nm was normalized to vehicle-treated controls and expressed as % of control. Bars represent mean ± S.D. from three independent experiments; overlaid points indicate individual replicates. Statistical significance is shown for SC18 co-treatment versus the corresponding single-agent condition: * *p* < 0.05, ** *p* < 0.01, *** *p* < 0.001.

To evaluate whether SC18 potentiates commonly used anti-tumor drugs, we performed MTT viability assays in the four melanoma lines. Cells were treated with doxorubicin (2 μM), Me344 (10 μM), PABA/NO (10 μM), paclitaxel (Taxol, 1 μM), or vinblastine (2 μM), either alone or in combination with SC18 (5 μM). Across the full drug panel, co-treatment with SC18 consistently reduced cell viability compared to the single-agent treatment, indicating enhanced anti-tumor potential (Figure 8). This sensitizing effect was more pronounced in the amelanotic SKMel28 and YUMM cells, while the melanotic MNT-1 and B16 cells showed comparatively attenuated responses. Collectively, these results support SC18 as a viable combinatorial drug, with the magnitudes of sensitization influenced by melanin pigmentation.

## 4. Discussion

Melanoma shows pronounced metabolic heterogeneity and plasticity, allowing tumor cells to flexibly use glycolysis and OXPHOS to support growth and survival [5,6]. Under MAPK-targeted therapy (e.g., BRAF/MEK inhibitors), many melanomas shift from glycolysis toward OXPHOS, promoting adaptive drug resistance [5,16]. This metabolic state is further shaped by the tumor microenvironment, which influences immune evasion and treatment response [5,6,8]. Elevated OXPHOS also correlates with aggressive disease (vertical growth, brain metastasis, and distant spread) and is often linked to enhanced glutamine use, PGC1α-driven programs, altered oxidative stress control, and reduced immune activation [17,18,19]. Although inhibiting OXPHOS can suppress tumor growth, direct OXPHOS inhibition has shown toxicities, highlighting the need for alternative strategies [16,18]. Accordingly, there is a strong rationale for identifying upstream mitochondrial regulators whose targeting may destabilize tumor bioenergetics while avoiding the liabilities associated with direct respiratory chain inhibition. In this context, our findings identify VDAC disruption as a potential strategy to interfere with melanoma metabolic plasticity at a functionally central control point.

VDAC isoforms function as regulators of mitochondrial metabolism by mediating metabolite transport, preserving membrane potential (Δψm), and coordinating apoptotic signaling [26,29]. Consistent with this central role, we observed high basal expressions of VDAC1 and VDAC3 across all melanoma cell lines examined. This finding is notable in light of our GEPIA2 analysis, which showed that VDAC1 and VDAC3 are upregulated in SKCM and are associated with poorer clinical outcome, supporting the idea that these isoforms may contribute functionally to melanoma progression. SC18 treatment reduced mitochondrial membrane potential, suppressed oxygen consumption rates, TCA cycle substrate utilization, and impaired ATP production, indicating that its cytotoxicity is mediated, at least in part, through VDAC antagonism. Because VDAC facilitates the exchange of respiratory substrates and adenine nucleotides across the outer mitochondrial membrane [20,21], its inhibition would be expected to impose a progressive bioenergetic constraint on tumor cells. This interpretation is consistent with previous reports showing that SC18 modulates NADH-dependent VDAC channel regulation and reduces mitochondrial membrane potential in hepatocarcinoma cells [23,24]. Our data extend those observations to melanoma and further indicate that the cellular consequences of VDAC inhibition are shaped by the metabolic state of the tumor subtype. Importantly, apoptosis analyses further support apoptotic cell death as a downstream consequence of SC18-induced mitochondrial disruption. Annexin V/PI flow cytometry showed that SC18 increased total Annexin V-positive populations, and caspase-3 activity assays confirmed increased apoptotic signaling across all four melanoma cell lines. These findings suggest that SC18-induced inhibition of melanoma cell survival is associated with mitochondrial dysfunction-linked apoptosis.

A defining downstream consequence of SC18-mediated mitochondrial disruption was the induction of oxidative stress. Across melanoma models, SC18 increased total intracellular ROS, elevated mitochondrial superoxide levels, and enhanced lipid peroxidation, indicating that mitochondrial redox buffering was substantially compromised following treatment. These effects are mechanistically coherent, as impaired metabolite exchange through VDAC [20,21] can reduce respiratory efficiency, promote electron leakage, and thereby amplify ROS generation. In turn, excessive ROS can further damage mitochondrial components, creating a feed-forward cycle of energetic failure and oxidative injury. Thus, oxidative stress in this context is not merely an associated phenotype, but likely an integral mediator of SC18-induced cytotoxicity. Although mitochondrial ROS, ER stress, and calcium dysregulation are interconnected stress pathways, ER stress was not the primary focus of the present study. Under the current experimental conditions, our preliminary data did not support ER stress as a dominant response to SC18 treatment. Therefore, the present findings support mitochondrial depolarization, oxidative stress, and apoptosis as the predominant responses to SC18, while ER stress and calcium-dependent signaling remain potential context-dependent mechanisms for future investigation.

The differential ROS responses between melanotic and low/amelanotic cells provide insight into the mode of action of SC18. Consistent with melanin quantification in the melanoma cell lines used in this study, MNT-1 cells contained the highest melanin level, B16 cells showed intermediate melanin content, whereas SKMel28 and YUMM cells had little to no detectable melanin. In line with this pigmentation pattern and the inherently greater antioxidant capacity associated with melanin-producing melanoma cells [12], melanotic MNT-1 and B16 cells showed relatively lower mitochondrial ROS accumulation following SC18 exposure than the low/amelanotic SKMel28 and YUMM cells. Melanin biosynthesis requires substantial metabolic investment and generates a microenvironment enriched in antioxidant intermediates [30,31,32], which may partially buffer SC18-induced oxidative stress. In contrast, amelanotic melanoma cells, characterized by lower intrinsic antioxidant reserves, exhibited more pronounced ROS accumulation and greater susceptibility to mitochondrial injury. These findings are consistent with prior studies showing that pigmentation status in melanoma is associated with distinct metabolic and redox states, including differences in antioxidant buffering, ATP production, and utilization of glycolysis, OXPHOS, and pentose phosphate pathway flux [12,14,27,28]. Our results therefore provide functional support for the concept that amelanotic melanoma cells occupy a more redox-vulnerable state and are less able to compensate when mitochondrial homeostasis is perturbed.

The increase in lipid ROS after SC18 treatment further raises the possibility that ferroptosis, or other lipid oxidation-associated cell death pathways, may contribute to SC18-induced cytotoxicity in susceptible subtypes [33]. Although ferroptosis was not directly assessed in the present study, this possibility is biologically plausible because lipid peroxide accumulation is a key feature of ferroptotic stress and has been linked to therapeutic vulnerability in melanoma [33,34]. The observed lipid ROS increase therefore suggests that SC18 may intersect not only with mitochondrial redox collapse, but also with downstream lipid peroxidation-dependent death pathways. Future studies using ferroptosis modulators will be important to define the extent to which this mechanism contributes to SC18 activity. In addition to ferroptosis, future studies should also examine whether SC18-mediated VDAC disruption affects autophagy and mitophagy, as these mitochondrial quality-control pathways may further shape melanoma cell responses to metabolic and redox stress.

The differences in IC_50_ values and dose–response curves among the melanoma sub-types underscore the importance of cellular metabolic state in influencing SC18 sensitivity. Amelanotic melanoma cells exhibited lower IC_50_ values, indicative of limited metabolic buffering and a reduced threshold for mitochondrial failure. These characteristics, combined with lower mitochondrial antioxidant reserves and greater reliance on OXPHOS-linked ATP production, rendered amelanotic cells more vulnerable to SC18-induced bioenergetic stress and oxidative damage. In contrast, melanotic MNT-1 and B16 cells had higher IC_50_ values, indicative of greater tolerance to SC18. Their moderate OXPHOS reliance, coupled with melanin-associated antioxidant defenses [30,31,32], likely partially compensates for VDAC inhibition and elevated ROS.

Consistently, SC18 also potentiated the cytotoxic effects of several conventional anticancer agents, including doxorubicin, Me344 [35], PABA/NO [36], taxol, and vinblastine, with more pronounced chemosensitization in amelanotic SKMel28 and YUMM cells than in melanotic MNT-1 and B16 cells. This may reflect a reduction in the metabolic reserve required for cellular adaptation, thereby lowering the threshold for therapy-induced lethality. Given the established role of metabolic plasticity in melanoma resistance, VDAC-directed mitochondrial destabilization may enhance the efficacy of existing therapies in metabolically susceptible melanoma subsets.

Beyond cytotoxicity, SC18 significantly impaired cell migration in wound-healing assays. Because cell motility is tightly coupled to mitochondrial ATP availability, cytoskeletal remodeling, and ROS-dependent signaling networks [37,38,39], the observed reduction in migratory capacity reflects the combined impact of SC18-induced mitochondrial dysfunction and redox stress. This anti-migratory activity suggests therapeutic value in targeting aggressive, invasive melanoma phenotypes that depend on metabolic plasticity for metastasis.

In summary, this study identifies SC18 as a potent mitochondrial disruptor that compromises melanoma cell viability by inducing bioenergetic collapse, promoting oxidative damage, triggering apoptotic cell death, and suppressing migratory capacity. The drug exhibits heightened activity in melanoma cells with high OXPHOS dependency and limited antioxidant capacity, highlighting its relevance for redox-vulnerable or metabolically defined melanoma subtypes. Further development of SC18 toward translational application will benefit from validation in three-dimensional melanoma models, patient-derived organoids, and in vivo melanoma models, together with evaluation of antitumor efficacy, toxicity, biodistribution, and potential synergy with ferroptosis inducers, mitochondrial inhibitors, immune checkpoint inhibitors, or conventional anticancer agents.

## Data Availability

The original contributions presented in this study are included in the article. Further inquiries can be directed to the corresponding authors.

## Abbreviations

The following abbreviations are used in this manuscript: AUC, area under the curve; DMEM, Dulbecco’s modified Eagle’s medium; ECAR, extracellular acidification rate; FBS, fetal bovine serum; H_2_DCFDA, 2′,7′-dichlorodihydrofluorescein diacetate; OCR, oxygen consumption rate; OXPHOS, oxidative phosphorylation; ROS, reactive oxygen species; TCA, tricarboxylic acid; TMRM, tetramethylrhodamine; VDACs, voltage-dependent anion channels.

## Appendix A

**Table A1:** Key resources table.

Reagent or Resource	Source	Identifier
**Antibodies**		
Anti-VDAC1/VDAC3	Abcam	Cat#AB14734
Anti-VDAC2	Abcam	Cat#AB37985
Anti-beta actin	Sigma	Cat#A5441
Anti-Mouse IRDye 680CW	LI-COR	Cat#926-68070
Anti-Mouse IRDye 800CW	LI-COR	Cat#926-32210
Anti-Rabbit IRDye 680CW	LI-COR	Cat#926-68071
Anti-Rabbit IRDye 800CW	LI-COR	Cat#926-32211
**Chemicals**		
VDAC inhibitor SC18S	Probechem	Cat#PC-38524
MTT (3-(4,5-Dimethylthiazol-2-yl)-2,5-Diphenyltetrazolium Bromide)	Sigma	M6494
Tetramethylrhodamine	Sigma	T668
MitoSOX™ Mitochondrial Superoxide Indicators	Thermo Fisher	M36008
BODIPY™ 650/665	Thermo Fisher	D10001
H2DCFDA	Thermo Fisher	D399
Oligomycin A	Sigma	O4876
Rotenone	Sigma	R8875
Antimycin A	Sigma	A8674
Doxorubicin	Sigma	D1515
Vinblastine	Sigma	PHR2698
Paclitaxel	Selleck Chemicals	S1150
PVDF Membrane, low fluorescence	Sigma	IPFL00010
Melanin	MP Biomedicals	MFCD00131581
**Critical commercial assays**		
Seahorse XFe96/XF Pro FluxPak	Agilent Technologies	103792-100
DMEM medium	Agilent Technologies	103575-100
Glucose solution	Agilent Technologies	103577-100
L-glutamine solution	Agilent Technologies	103579-100
Sodium pyruvate solution	Agilent Technologies	103578-100
Pierce BCA protein assay kit	Thermo Fisher	23225
FITC Annexin V Apoptosis Detection Kit I	BD Pharmingen™	556547
EnzChek Caspase-3 Assay Kit	Invitrogen	MP 13184
**Experimental models: cell lines**		
B16-F1	ATCC	Cat#CRL-6323
YUMM	ATCC	Cat#CRL-3362
MNT-1	ATCC	Cat#CRL-3450
SKMel28	Prof. P H. Howe, MUSC	N/A
**Software and algorithms**		
GraphPad Prism v8	GraphPad	N/A
CytExpert v2.1	Beckman	N/A

N/A: Not applicable.
